# Delayed Presentation and Mortality in Children With Sepsis in a Public Tertiary Care Hospital in Tanzania

**DOI:** 10.3389/fped.2021.764163

**Published:** 2021-11-30

**Authors:** Audrey Marilyn Smith, Hendry R. Sawe, Michael A. Matthay, Brittany Lee Murray, Teri Reynolds, Teresa Bleakly Kortz

**Affiliations:** ^1^Institute for Global Health Sciences, University of California, San Francisco, San Francisco, CA, United States; ^2^Department of Emergency Medicine, Muhimbili University of Health and Allied Sciences, Dar es Salaam, Tanzania; ^3^Departments of Medicine and Anesthesia, Cardiovascular Research Institute, University of California, San Francisco, San Francisco, CA, United States; ^4^Department of Pediatrics and Department of Emergency Medicine, Emory University School of Medicine, Atlanta, GA, United States; ^5^World Health Organization, Geneva, Switzerland; ^6^Division of Critical Care, Department of Pediatrics, University of California, San Francisco, San Francisco, CA, United States

**Keywords:** pediatric sepsis, pediatric critical care, global health, pediatric emergency medicine, sub-Saharan Africa, health disparities, resource-limited

## Abstract

**Background:** Over 40% of the global burden of sepsis occurs in children under 5 years of age, making pediatric sepsis the top cause of death for this age group. Prior studies have shown that outcomes in children with sepsis improve by minimizing the time between symptom onset and treatment. This is a challenge in resource-limited settings where access to definitive care is limited.

**Methods:** A secondary analysis was performed on data from 1,803 patients (28 days−14 years old) who presented to the emergency department (ED) at Muhimbili National Hospital (MNH) from July 1, 2016 to June 30, 2017 with a suspected infection and ≥2 clinical systemic inflammatory response syndrome criteria. The objective of this study was to determine the relationship between delayed presentation to definitive care (>48 h between fever onset and presentation to the ED) and mortality, as well as the association between socioeconomic status (SES) and delayed presentation. Multivariable logistic regression models tested the two relationships of interest. We report both unadjusted and adjusted odds ratios and 95% confidence intervals.

**Results:** During the study period, 11.3% (*n* = 203) of children who presented to MNH with sepsis died inhospital. Delayed presentation was more common in non-survivors (*n* = 90/151, 60%) compared to survivors (*n* = 614/1,353, 45%) (*p* ≤ 0.01). Children who had delayed presentation to definitive care, compared to those who did not, had an adjusted odds ratio for mortality of 1.85 (95% CI: 1.17–3.00).

**Conclusions:** Delayed presentation was an independent risk factor for mortality in this cohort, emphasizing the importance of timely presentation to care for pediatric sepsis patients. Potential interventions include more efficient referral networks and emergency transportation systems to MNH. Additional clinics or hospitals with pediatric critical care may reduce pediatric sepsis mortality in Tanzania, as well as parental education programs for recognizing pediatric sepsis.

## Introduction

Sepsis is a clinical syndrome defined as a systemic inflammatory response associated with an infection (1). If untreated, sepsis can lead to septic shock, a condition that can result in multi-organ system failure and death (2, 3). Over 40% of the global burden of sepsis occurs in children under 5 years of age, with 20.3 million cases in 2017, causing the highest burden of mortality for this age group (2.9 million deaths in 2017) (4, 5).

Sepsis is the common final pathway for most infectious disease-related deaths, indicating that regions with higher burdens of pediatric communicable diseases have higher burdens of sepsis, such as South-East Asia and sub-Saharan Africa (4, 6, 7). Pediatric sepsis data are sparse in low- and middle-income countries (LMICs), making it difficult to assess trends in pediatric sepsis cases and mortality. Health facilities in LMICs sometimes lack the resources necessary to recognize and treat pediatric sepsis, such as sufficient intensive care unit beds, monitoring devices, medications, or health professionals trained in pediatric emergency or critical care (8–12). The lack of data on sepsis is a barrier to improving pediatric outcomes in resource-limited settings.

Basic acute and critical care and evidence-based therapies such as antibiotics and fluid resuscitation have been shown to reduce the likelihood of negative outcomes for septic children (1, 4, 6, 8, 13–17), and longer treatment delays result in higher morbidity and mortality (8, 13–19). Current pediatric sepsis guidelines recommend immediate (within 1 h) administration of antibiotics, because delayed treatment with antibiotics is an independent predictor of mortality and organ dysfunction (13, 14, 18). Familial SES has also been shown to influence pediatric sepsis outcomes as well as delayed presentation to care (6). However, most studies on pediatric sepsis in resource-limited settings focus on timely administration of treatment within the hospita**l** rather than delays in arrival.

The objective of this secondary analysis was to assess SES as a possible risk factor for delayed presentation to definitive care, as well as the association between delayed presentation and mortality within this pediatric sepsis cohort.

## Materials and Methods

### Study Design

**Figure 1:**
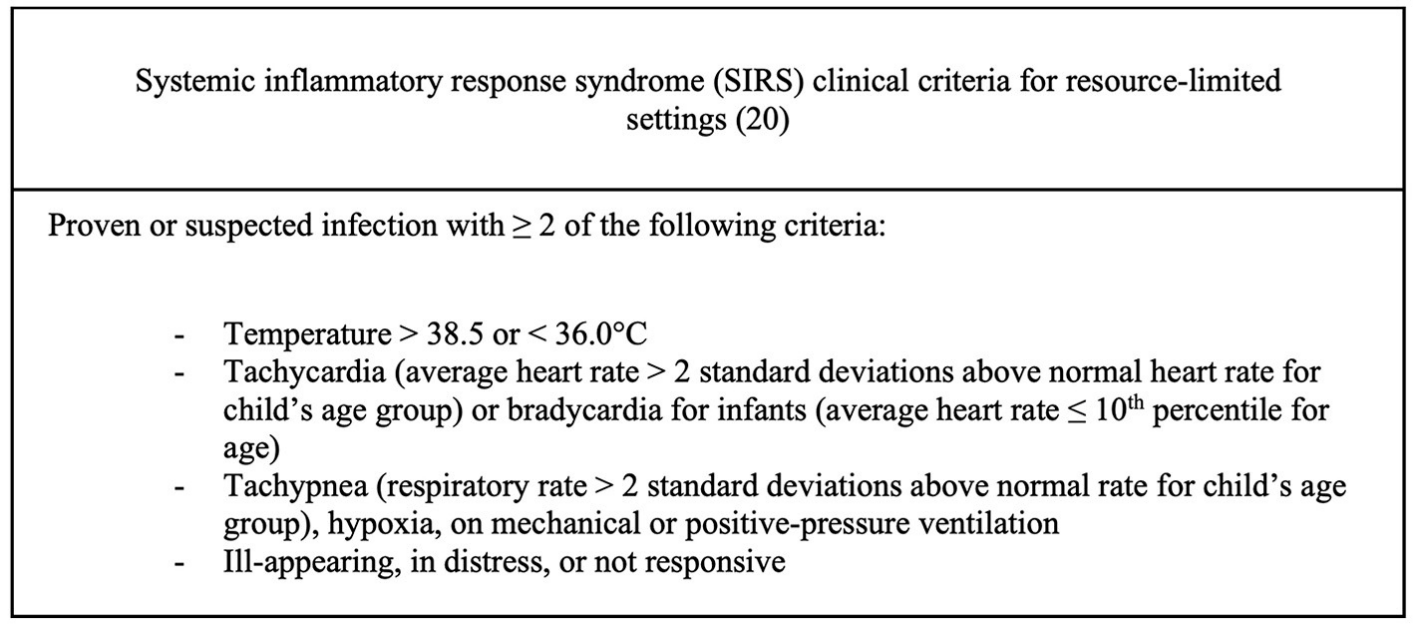
SIRS criteria.

This is a secondary analysis of a prospective cohort study conducted at Muhimbili National Hospital (MNH) in Tanzania. The study population was children 28 days to 14 years of age who presented to the ED of MNH over 12 months from July 1, 2016 to June 30, 2017. Eligible children were screened for suspected infection and at least two of the criteria for Systemic Inflammatory Response Syndrome (SIRS) adapted for resource-limited settings (Figure 1) (20).

**Figure 2:**
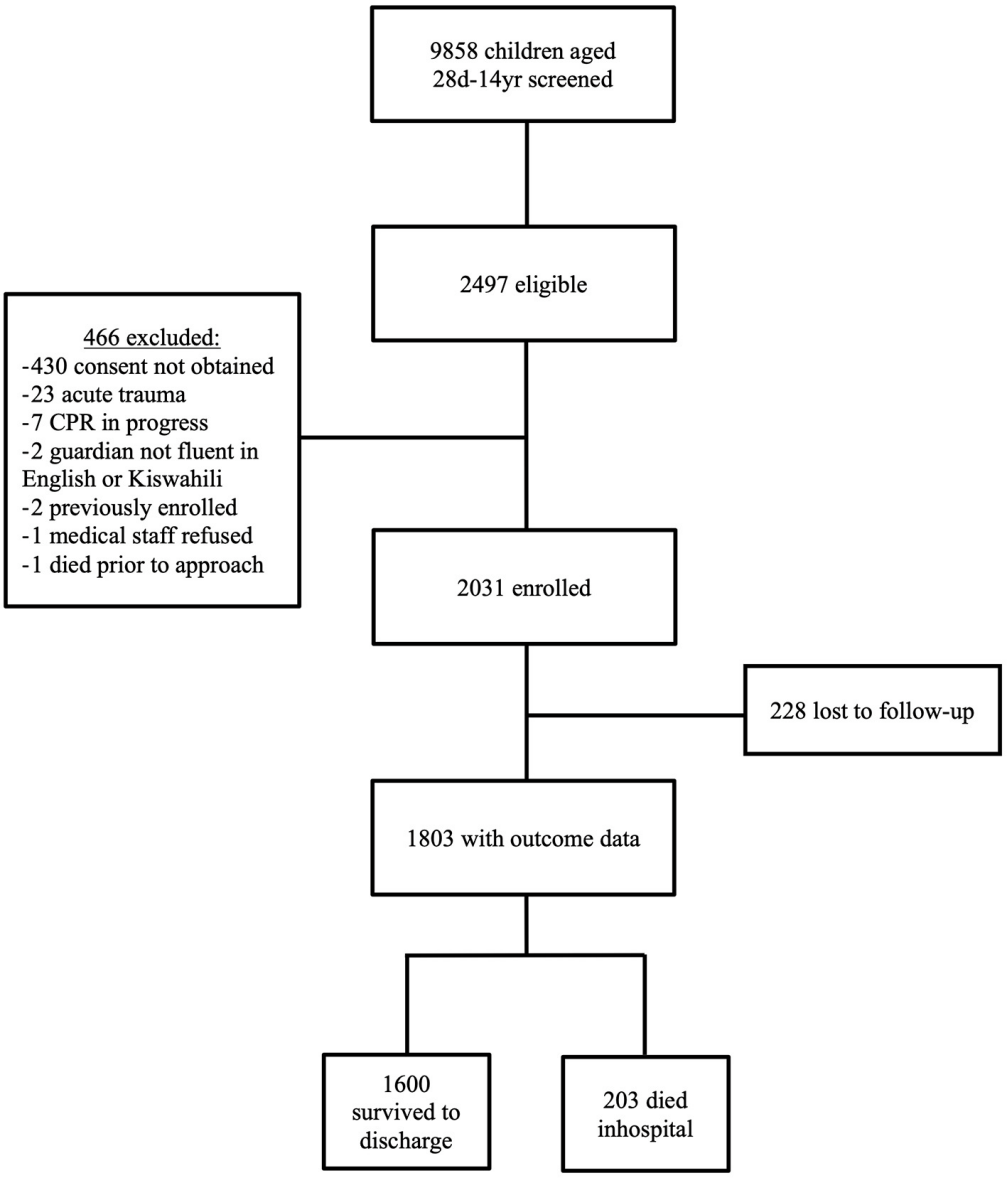
Flow-chart depicting enrollment for this study, including criteria for exclusion, children lost to follow-up, and mortality outcomes.

Children were excluded for lack of consent, acute trauma, active cardiopulmonary resuscitation (CPR), lack of English of Kiswahili fluency of the guardian, and death prior to approach. In total, 2,031 children were included in the study, and 1,803 (88.8%) had outcome data (Figure 2).

### Study Site

The National Hospital at Muhimbili University of Health and Allied Sciences is a public, tertiary-level referral hospital located in Dar es Salaam, Tanzania. At the time of the study, MNH had the only 24 h, public ED in the country. MNH receives hundreds of pediatric sepsis cases each month, and patients travel from every region of Tanzania, some over 1,000 km, for definitive care. While there are other emergency departments in Tanzania that can provide aspects of definitive care such as antibiotics and fluid resuscitation, MNH is the only public hospital with pediatric subspecialty care, meaning it has pediatricians with advanced training in emergency medicine. With respect to resources, it is one of the few hospitals in Tanzania equipped to provide emergency and critical care to children (21).

### Data Collection and Management

Research personnel collected data from electronic medical records, paper charts, care providers, and guardians during the study period (22). Data were entered into and managed using REDCap (Research Electronic Data Capture) tools version 7.2.2 hosted at MNH. Data were deidentified prior to analysis. REDCap is a secure, web-based application designed to support data capture for research studies, providing (1) an intuitive interface for validated data entry; (2) audit trails for tracking data manipulation and export procedures; (3) automated export procedures for seamless data downloads to common statistical packages; and (4) procedures for importing data from external sources.

### Statistical Analysis

All statistical analyses were performed using R Statistical Software (version 1.4.1717; R Foundation for Statistical Computing, Vienna, Austria, 2021). Descriptive statistics assessed patient baseline characteristics including pertinent demographic data, proxies for SES, clinical characteristics, and hospital pre-arrival information, such as mode of transport to the hospital and fever duration (Tables 1–**3**). Univariate statistics were generated to assess these characteristics in the full cohort. Bivariate tests compared survivors and non-survivors using Fisher's exact tests for categorical variables with skewed distributions and chi-squared tests for categorical variables with normal distributions. Wilcoxon rank-sum tests compared the medians of continuous variables with skewed distributions. *P*-values ≤ 0.05 were considered statistically significant.

**Table 1:** Descriptive statistics for the full cohort and a comparison of demographic characteristics by mortality outcome.

**Demographic characteristic**	**Total**	**Survivors**	**Non-survivors**	***P*-value**
	***N* = 1803**	**(*n* = 1,600)**	**(*n* = 203)**	
Age, months, median (IQR)	24.9 (13.1–53.1)	25.8 (13.8–54.6)	17.1 (7.7–37.0)	*P* < 0.001
Age, *n* (%)				
<2 years	854 (47.4)	732 (45.8)	122 (60.1)	*P* < 0.001
2–5 years	540 (30.0)	498 (31.1)	42 (20.7)	*p* ≤ 0.01
>5 years	409 (22.7)	370 (23.1)	39 (19.2)	*p* = 0.24
Male sex, *n* (%)	1033 (57.3)	920 (57.5)	113 (55.7)	*p* = 0.67
Regional address, *n* (%)				
Dar es Salaam	1394 (77.3)	1237 (77.3)	157 (77.3)	*p* = 1.00
Neighboring regions	240 (13.3)	210 (13.1)	30 (14.8)	*p* = 0.59
Mid-distance regions	97 (5.4)	88 (5.5)	9 (4.4)	*p* = 0.64
Far regions	72 (4.0)	65 (4.1)	7 (3.4)	*p* = 0.82
Malaria positive, *n* (%)	99/1429 (6.9)	89/1264 (7.0)	10/165 (6.1)	*p* = 0.76
HIV positive, *n* (%)	22/229 (9.6)	15/188 (8.0)	7/41 (17.1)	*p* = 0.13
Fully immunized, *n* (%)	1770/1792 (98.8)	1575/1591 (99.0)	195/201 (97.0)	*p* = 0.04
Malnourished, *n* (%):				
Underweight	469/1615 (29.0)	398/1435 (27.7)	71/180 (39.4)	*P* ≤ 0.001
Wasting	344/1318 (26.1)	296/1173 (25.2)	48/145 (33.1)	*p* = 0.05
Stunting	564/1544 (36.5)	493/1371 (36.0)	71/173 (41.0)	*p* = 0.22
Comorbidities, *n* (%)				
Anemia	20 (1.1)	17 (1.1)	3 (1.5)	*p* = 0.86
Asthma	31 (1.7)	27 (1.7)	4 (2.0)	*p* = 1.00
Cancer	28 (1.6)	24 (1.5)	4 (2.0)	*p* = 0.83
Cerebral palsy	85 (4.7)	74 (4.6)	11 (5.4)	*p* = 0.74
Congenital anomalies	11 (0.6)	10 (0.6)	1 (0.5)	*p* = 1.00
Congenital heart disease	173 (9.6)	142 (8.9)	31 (15.3)	*p* = 0.01
Downs syndrome	13 (0.7)	11 (0.7)	2 (1.0)	*p* = 0.97
Hydrocephalus	21 (1.2)	18 (1.1)	3 (1.5)	*p* = 0.93
Seizure disorder	41 (2.3)	39 (2.4)	2 (1.0)	*p* = 0.29
Sickle cell anemia	110 (6.1)	108 (6.8)	2 (1.0)	*P* < 0.001
Tuberculosis	25 (1.4)	17 (1.1)	8 (3.9)	*P* < 0.001
Other	73 (4.0)	63 (3.9)	10 (4.9)	*p* = 0.65
≥1 comorbidity, *n* (%)	585 (32.4)	506 (31.6)	79 (38.9)	*p* = 0.04

*IQR, interquartile range; HIV, Human Immunodeficiency Virus*.

Delayed presentation to definitive care was determined by measuring the time between onset of fever to the time the child reached the ED of MNH, as reported by the guardian. The definition for delayed presentation to definitive care was a fever duration >48 h from fever onset to arrival at the ED.

The proportion of children that were underweight, had wasting, and had stunting were calculated based on the WHO's official guidelines for child anthropometry (Table 1) (23). The R package *zscorer* (version 0.3.1) was used to calculate *z*-scores but was limited to weight-for-age *z*-scores for children under 120 months, weight-for-height *z*-scores for children between 65 and 120 cm, and height-for-age *z*-scores for children between 24 and 228 months. For the first logistic regression model, wasting was used to represent malnutrition as it was determined to be the most clinically significant form of malnutrition for this cohort.

**Figure 3:**
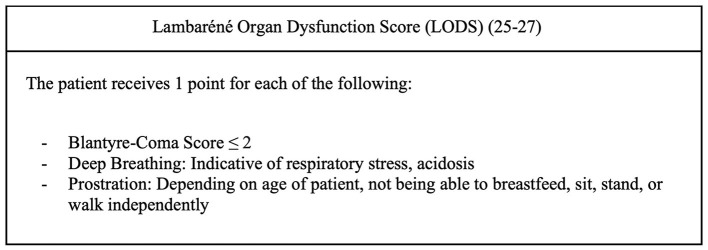
LODS criteria.

Potential confounding factors in the relationship between delayed presentation and mortality were identified *a priori* based on clinical knowledge and a literature review, which were severity of disease (measured by the Lambaréné Organ Dysfunction Score, LODS, Figure 3), age, patient comorbidities, and malnutrition (1, 13, 14, 24–32). Patient age was considered because there are age-associated differences in clinical presentations of sepsis that could potentially influence time to presentation to care (1, 28), and age is a well-known predictive factor in pediatric sepsis mortality (24, 29). Comorbidities among patients in the cohort were also considered in the analysis because they have been found to influence pediatric sepsis mortality (13, 14). Malnutrition was considered a standalone comorbidity in analysis, as it is significantly associated with pediatric sepsis mortality (30–32) and could affect time to presentation to care.

The association between SES and delayed presentation to definitive care was also explored. Potential confounders in this relationship were maternal literacy and patient region of origin in Tanzania, also identified *a priori* based on a literature review (33, 34). Maternal literacy represented parental education level, which is related to SES and could also impact caregiver care-seeking behavior (33). Region of origin was considered because children may have had to travel long distances to reach MNH for definitive care, and because Tanzania's many regions differ in average SES (34).

Proxies for SES were collected for this cohort and an ownership score (range: 0–3) was generated based on the reported presence of three variables: household electricity, in-home flush/pour toilet, and access to an improved water source. An improved water source is defined by the WHO as sources that are “protected from outside contamination, particularly fecal matter” (35).

The first logistic regression model generated unadjusted and adjusted odds ratios (OR) and respective 95% confidence intervals (95% CI) for the relationship between delayed presentation and mortality. The second logistic regression model explored the association between the SES of the participants, using the ownership score, and delayed presentation to care, generating unadjusted and adjusted ORs and respective 95% CIs.

### Ethics Approval Statement

This study was carried out in accordance with the recommendations and approval of the Institutional Review Boards and Committees on Human Research at Muhimbili University of Health and Allied Sciences (Ref. No. 2016-03-30/AEC/Vol.X/201) and the University of California, San Francisco (IRB # 16-18977, Ref. No. 161295). Written, informed consent from all guardians and assent from subjects when appropriate was obtained in accordance with the Declaration of Helsinki.

## Results

### Baseline Characteristics: Demographics

Overall, there was an in-hospital mortality rate of 11.3%, with 1,600 survivors and 203 non-survivors. The median age of children enrolled in the study was 25 months (IQR 13–54 months) with 77.3% (*n* = 1,394) of the children under 5 years of age (Table 1). Of the study population with outcome data, 1,394 patients (77.3%) were from the Dar es Salaam region of Tanzania.

Out of the patients tested, 6.9% (*n* = 99/1,429) were positive for malaria and 9.6% (*n* = 22/229) were HIV-positive. Based on WHO child anthropometry guidelines (23), 29.0% (*n* = 469/1,615) of patients were underweight, 26.1% (*n* = 344/1,318) had wasting, and 36.5% (*n* = 564/1,544) had stunting. Malnutrition was the most common comorbidity in the cohort, followed by congenital heart disease (*n* = 173, 9.6%) and sickle cell anemia (*n* = 110, 6.1%) (Table 1).

There were key differences noted in several baseline demographic characteristics between survivors and non-survivors (Table 1). Non-survivors were of a younger median age (17.1 months, IQR: 7.7–37.0) upon presentation, compared to survivors (25.8 months, IQR: 13.8–54.6) (*p* < 0.001). At the time of arrival to the hospital, more of the non-survivors were underweight (*n* = 71/180, 39.4%), compared to the survivors (*n* = 398/1,435, 27.7%) (*p* ≤ 0.001). Similarly, more non-survivors had wasting (*n* = 48/145, 33.1%), compared to survivors (*n* = 296/1,173, 25.2%) (*p* = 0.05). More of the non-survivors had at least one significant comorbidity (*n* = 79, 38.9%), compared to the survivors (*n* = 506, 31.6%) (*p* = 0.04).

### Baseline Characteristics: SES

The highest level of education reached by the patients' mothers differed; while 376 (23.5%) of the survivors' mothers held a university of other advanced degree, this was true for only 12 of the non-survivors (5.9%) (*p* < 0.001) (Table 2). When guardians were asked if their homes had electricity, less guardians of non-survivors answered affirmatively (*n* = 146/200, *n* = 73.0%), compared to the guardians of the survivors (*n* = 1,253/1,596, 78.5%) (*p* = 0.04). Congruently, fewer non-survivors had a flush/pour toilet in their homes (*n* = 106, 52.2%), compared to survivors (*n* = 993, 62.1%) (*p* = 0.01).

**Table 2:** Descriptive statistics for the full cohort and a comparison of socioeconomic characteristics by mortality outcome.

**Characteristic**	**Total**	**Survivors**	**Non-survivors**	***P*-value**
	***N* = 1803**	**(*n* = 1,600)**	**(*n* = 203)**	
Maternal literacy, *n* (%)	1694/1798 (94.2)	1507/1595 (94.5)	187 (92.1)	*p* = 0.23
Maternal education level, *n* (%)				
No formal school	87 (4.8)	74 (4.6)	13 (6.4)	*p* = 0.35
Primary school	870 (48.3)	740 (46.3)	130 (64.0)	*P* < 0.001
Secondary school	434 (24.1)	390 (24.4)	44 (21.7)	*p* = 0.45
University/advanced degree	388 (21.5)	376 (23.5)	12 (5.9)	*P* < 0.001
Unknown	24 (1.3)	20 (1.3)	4 (2.0)	*p* = 0.60
No. of children <18 years in household, median (IQR)	2 (1–3)	2 (1–3)	2 (1–3)	*p* = 0.58
No. <5 years, median (IQR)	1 (1–2)	1 (1–2)	1(1–2)	*p* = 0.36
Electricity in home, *n* (%)	1399/1796 (77.9)	1253/1596 (78.5)	146/200 (73.0)	*p* = 0.04
Toilet in home, *n* (%)	1099 (61.0)	993 (62.1)	106 (52.2)	*p* = 0.01
Improved water source, *n* (%)	1715 (95.1)	1521 (95.1)	194 (95.6)	*p* = 0.89
Private tap	505 (28.0)	475 (29.7)	30 (14.8)	*P* < 0.001
Public tap or standpipe	1065 (59.1)	915 (57.2)	150 (73.9)	*P* < 0.001
Tube well or borehole	227 (12.6)	201 (12.6)	26 (12.8)	*p* = 1.00
Protected spring	7 (0.4)	6 (0.4)	1 (0.5)	*p* = 1.00

*No., number; IQR, interquartile range*.

### Baseline Characteristics: Hospital Pre-arrival and Illness Severity

The hospital pre-arrival baseline characteristics revealed that 42.4% percent (*n* = 678/1,598) of the cohort received antibiotics before presentation to MNH, and 77.3% (*n* = 836/1,081) had been referred by another hospital or clinic (Table 3).

**Table 3:** Descriptive statistics for the full cohort and a comparison of pre-arrival characteristics and illness severity measures by mortality outcome.

**Characteristic**	**Total**	**Survivors**	**Non-survivors**	***P*-value**
	***N* = 1803**	**(*n* = 1,600)**	**(*n* = 203)**	
Fever duration, *n* (%)				
≤ 48 h	800 (44.4)	739 (46.2)	61 (30.0)	*P* ≤ 0.001
>48 h	704 (39.0)	614 (38.4)	90 (44.3)	*p* = 0.12
Unknown	299 (16.6)	247 (15.4)	52 (25.6)	*p* = 0.00
Antibiotics pre-arrival, *n* (%)	352/833 (42.3)	269/675 (39.9)	83/158 (52.5)	*p* = 0.01
Referred by hospital or clinic, *n* (%):	836/1081 (77.3)	678/1598 (42.4)	158/203 (77.8)	*P* < 0.001
Transportation method, *n* (%)				
Ambulance	517 (28.7)	380 (23.8)	137 (67.5)	*P* < 0.001
Bus	783 (43.4)	739 (46.2)	44 (21.7)	*P* < 0.001
Private car	369 (20.5)	353 (22.1)	16 (7.9)	*P* < 0.001
Taxi	50 (2.8)	48 (3.0)	2 (1.0)	*p* = 0.16
Walked	55 (3.1)	54 (3.4)	1 (0.5)	*p* = 0.04
Other	23 (1.3)	20 (1.3)	3 (1.5)	*p* = 1.00
Unknown	7 (0.4)	7 (0.4)	0	*p* = 0.73
SIRS criteria *n* (%)				
Abnormal respiratory rate	1614 (89.5)	1420 (88.8)	194 (95.6)	*p* = 0.00
Abnormal heart rate	1001 (55.5)	878 (54.9)	123 (60.6)	*p* = 0.13
Ill appearing, in distress, not responsive	1115 (61.8)	950 (59.4)	165 (81.3)	*P* < 0.001
LODS, *n* (%)				
0	893 (49.5)	823 (51.4)	70 (34.5)	*P* < 0.001
1	717 (39.8)	629 (39.3)	88 (43.3)	*p* = 0.30
2	171 (9.5)	140 (8.8)	31 (15.3)	*P* ≤ 0.01
3	22 (1.2)	8 (0.5)	14 (6.9)	*P* < 0.001
AVPU scale, *n* (%)				
Alert	1616 (89.6)	1479 (92.4)	137 (67.5)	*P* < 0.001
Responds to verbal	30 (1.7)	22 (1.4)	8 (3.9)	*p* = 0.02
Responds to pain	112 (6.2)	65 (4.1)	47 (23.2)	*P* < 0.001
Unresponsive	45 (2.5)	34 (2.1)	11 (5.4)	*p* = 0.01

*SIRS, systemic inflammatory response syndrome; LODS, Lambaréné Organ Dysfunction Score; AVPU, Alert-Verbal-Painful-Unresponsive*.

Survivors and non-survivors differed in fever duration, with non-survivors having more delayed presentation to definitive care than the survivors (*p* ≤ 0.001) (Table 3). More of the non-survivors received antibiotics before arriving (*n* = 83/158, 52.5%), compared to survivors (*n* = 269/675, 39.9%) (*p* = 0.01), and more non-survivors were referred to MNH by another clinic or hospital (*n* = 158/203, 77.8%), compared to survivors (*n* = 678/1,598, 42.4%) (*p* < 0.001).

**Figure 4:**
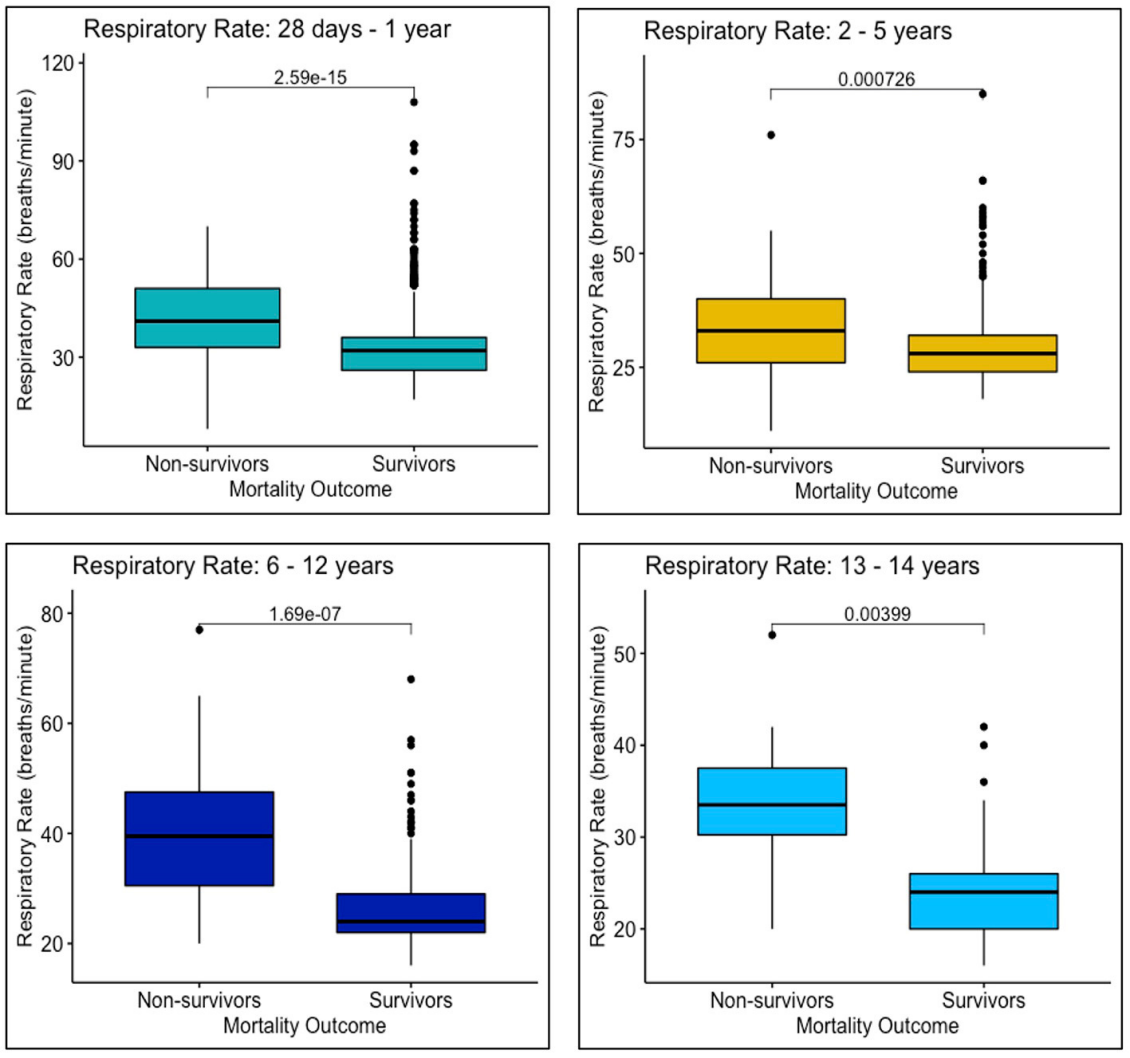
Respiratory rates upon arrival to MNH of survivors and non-survivors in each age group: 28 days−1 year, 2–5 years, 6–12 years, and 13–14 years.

**Figure 5:**
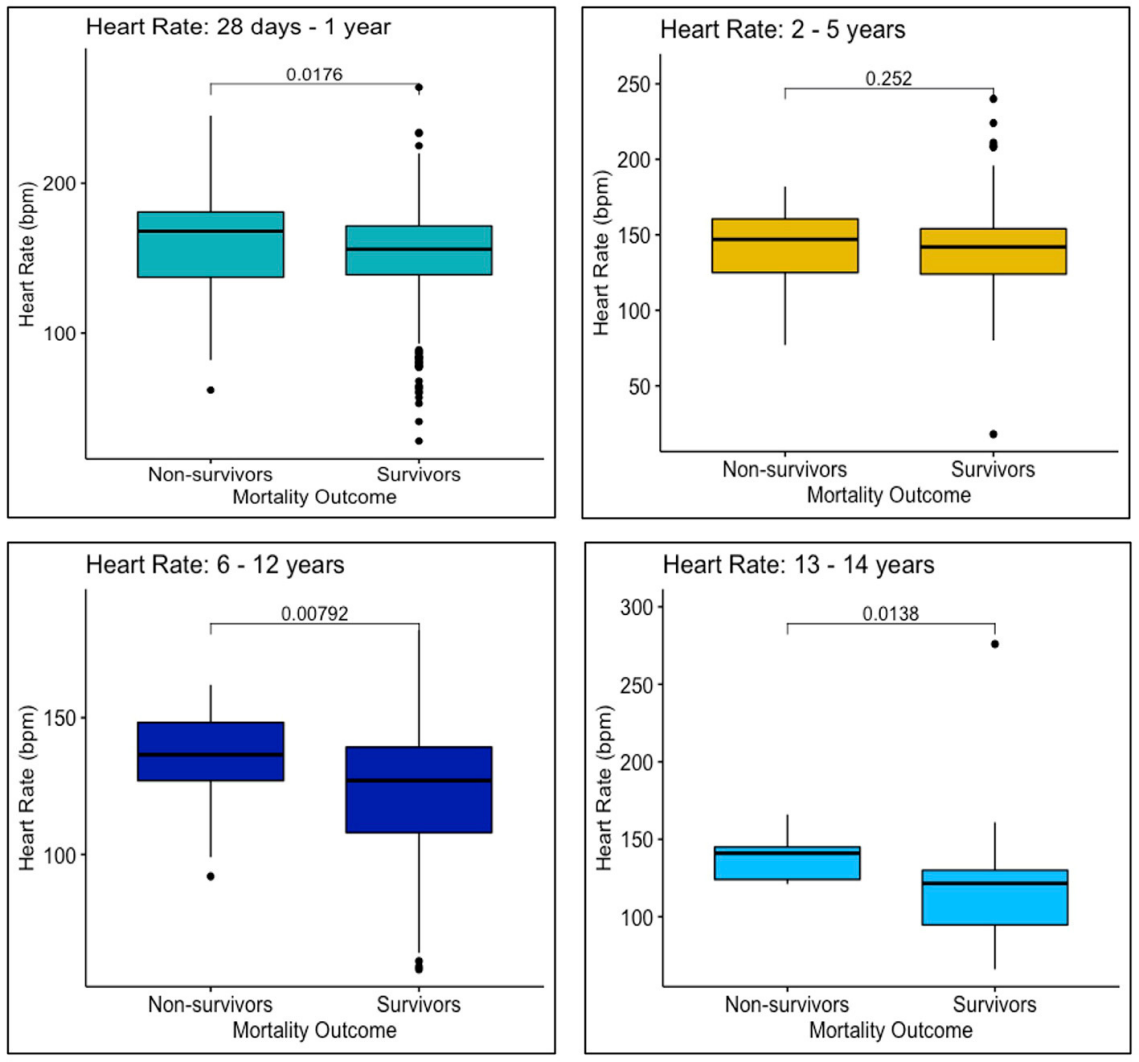
Heart rates upon arrival to MNH of survivors and non-survivors in each age group: 28 days−1 year, 2–5 years, 6–12 years, and 13–14 years.

Survivors and non-survivors differed in SIRS criteria met upon arrival, with a larger proportion of non-survivors presenting with abnormal respiratory rates (*n* = 194, 95.6%) compared to survivors (*n* = 1420, 88.8%) (*p* ≤ 0.00) (Table 3). Additionally, more non-survivors arrived appearing ill, in distress, or non-responsive (*n* = 165, 81.3%), compared to survivors (*n* = 950, 59.4%) (*p* < 0.001). Respiratory rates were higher among non-survivors for all age groups, compared to survivors of the same age groups (28 days−1 year: *p* < 0.001; 2–5 years: *p* < 0.001, 6–12 years: *p* < 0.001, 13–14 years: *p* ≤ 0.00) (Figure 4). Heart rates were significantly higher for non-survivors among all age groups except 2–5 years (28 days−1 year: *p* = 0.02; 2–5 years: *p* = 0.25; 6–12 years: *p* = 0.01; 13–14 years: *p* = 0.01) (Figure 5).

The LOD scores of survivors and non-survivors (range: 0–3) also differed significantly, with significantly less non-survivors scoring a 0 (*n* = 823, 51.4%) compared to survivors (*n* = 70, 34.5%) (*p* < 0.001), indicating greater severity of illness among non-survivors at the time of presentation. Congruently, as indicated by the Alert-Verbal-Painful-Unresponsive (AVPU) categorization, fewer non-survivors were alert upon their arrival to MNH (*n* = 137, 67.5%), compared to non-survivors (*n* = 1,479, 92.4%) (*p* < 0.001) (Table 3).

### Logistic Regression Models

The children who had delayed presentation to definitive care had an unadjusted OR for mortality of 1.75 (95% CI: 1.19–2.61) and an adjusted OR for mortality of 1.85 (95% CI: 1.17–3.00) compared to children who did not have delayed presentation to care (Table 4). The ownership variable was not significantly associated with delayed presentation after adjusting for maternal literacy and Tanzanian region of origin classification (Table 5). Comparing households with 3/3 ownership variables to those with 0/3, there was an unadjusted OR for delayed presentation of 0.43 (95% CI: 0.19–0.86) and an adjusted OR of 0.60 (95% CI: 0.26–1.27).

**Table 4:** Adjusted and unadjusted odds ratios and 95% confidence intervals for mortality and its association with delayed presentation.

**Variable**	**Unadjusted odds ratio**	**Unadjusted confidence interval**	**Adjusted odds ratio**	**Adjusted confidence interval**
**Delayed presentation**	1.75	1.19–2.61	1.85	1.17–3.00
Age (years)	0.95	0.90–1.00	0.72	0.61–0.82
Malnourished (wasting)	1.46	1.00–2.10	1.41	0.90–2.16
≥1 comorbidity	1.38	1.02–1.86	1.69	1.10–2.57
**Severity of Illness**				
LODS 0	Ref		Ref	
LODS 1	1.64	1.18–2.29	1.47	0.95–2.29
LODS 2	2.60	1.63–4.09	1.90	0.97–3.57
LODS 3	20.58	8.52–53.10	9.07	2.05–40.17

*LODS, Lambaréné Organ Dysfunction Score; Ref, reference group; Comorbidities included anemia, asthma, cancer, cerebral palsy, congenital anomalies, congenital heart disease, diabetes, Down syndrome, Human Immunodeficiency Virus (HIV), hydrocephalus, renal disease, seizure disorders, sickle cell anemia, tuberculosis, and other significant comorbidities*.

**Table 5:** Adjusted and unadjusted odds ratios and 95% confidence intervals for delayed presentation given number of ownership variables as a measure of SES.

**Variable**	**Unadjusted odds ratio**	**Unadjusted confidence interval**	**Adjusted odds ratio**	**Adjusted confidence interval**
SES (total no. ownership variables)				
0	Ref			
1	0.61	0.27–1.28	0.72	0.31–1.53
2	0.48	0.21–0.98	0.64	0.27–1.37
3	0.43	0.19–0.86	0.6	0.26–1.27
Maternal Literacy	0.64	0.38–1.03	0.81	0.48–1.35
Region of origin				
Dar es Salaam	Ref			
Neighboring	1.61	1.17–2.23	1.42	1.01–2.03
Mid-distance	1.55	0.96–2.58	1.38	0.84–2.33
Far	1.53	0.88–2.77	1.34	0.76–2.47

*SES, socioeconomic status; No., number; Ownership variables, in-home flush/pour toilet, household electricity, and access to an improved water source; Ref, reference group; Neighboring regions: Pwani, Mjini Magharibi, Unguja, Pemba, Tanga, and Morogoro; Mid-distance regions: Arusha, Dodoma, Iringa, Kilimanjaro, Lindi, Manyara, Mtwara, and Ruvuma; Far regions: Mbeya, Mwanza, Mara, Njombe, Kagera, Katavi, Kigoma, Geita, Rukwa, Singida, Shinyanga, Simiyu, and Tabora*.

## Discussion

### Key Findings

Out of the children enrolled in this study with outcome data, 11% did not survive to hospital discharge. Approximately half of the patients with available fever duration data presented to definitive care at MNH after the 48 h cut-off, and delayed presentation to definitive care was more common in non-survivors compared to survivors. A significantly larger proportion of non-survivors fulfilled certain SIRS, LODS, and AVPU criteria, three measures of illness severity, and non-survivors presented with more abnormal respiratory and heart rates than survivors. A logistic regression model confirmed that the children with delayed presentation to definitive care, compared to children who did not have delayed presentation, had 1.85 the odds of dying (CI: 1.17–3.00), after controlling for potential confounders. SES, measured by number of ownership variables, was not independently associated with delayed presentation.

### Interpretation of Findings

The 11% mortality among children with sepsis at MNH is consistent with other published pediatric sepsis cohort studies. A study that took place in Mbarara, Uganda with children ages 6 months to 5 years with a suspected or proven infection and a lower severity of illness at the time of presentation observed a 5% probability of inhospital mortality (36). Mortality in the Sepsis Prevalence, Outcomes, and Therapies (SPROUT) study, a global point prevalence study of severe sepsis, was 25%, but included subjects that met at least two SIRS criteria *and* had dysfunction of the cardiovascular system or two other organs, or acute respiratory distress syndrome (6). Most similar to the MNH cohort was the Fluid Expansion as Supportive Therapy (FEAST) study cohort from sites in Uganda, Kenya, and Tanzania, which observed a 7.3–10.6% mortality (depending on the treatment arm) within 48 h in non-malnourished children ages 60 days−12 years with severe febrile illness (37).

Notably, a study conducted in 2020 in the newly established pediatric intensive care unit at MNH, observed that 14% of pediatric deaths were from septicemia (38). This study concluded that the quality of intensive care for children “achieved the minimum acceptable standards” and would benefit from improving pediatric critical care training, hospital infrastructure, emergency equipment, and treatment protocols (38), which suggests that improvements could be made in reducing pediatric sepsis mortality at MNH.

Delayed presentation was a significant risk factor for mortality in this cohort, emphasizing the importance of timely presentation to definitive care at MNH for pediatric sepsis patients. MNH has noted many delays in its referral system, attributed to either patient caregiver behavior or delays from referral hospitals (38). Because more of the children who were referred to MNH from other lower-level facilities died, delays in sepsis recognition and referral at these facilities may be a contributor. A next step could be increasing education of providers at all levels of care, especially among primary providers, on pediatric sepsis recognition, timely initiation of appropriate treatment within the constraints of available resources, and sepsis cases that warrant referral. This increased provider education would be especially important in regions far from Dar es Salaam, where MNH is located. Such an intervention could be cost-effective, and a dedicated study designed to determine cost-effectiveness would be informative.

Because minimizing delays can be lifesaving for septic children, another potential intervention could be an educational program directed at caregivers teaching early warning signs and symptoms of sepsis. Parental education has been shown to be a successful tool for preventing progression to severe illness for certain pediatric conditions, for example, extreme hyperbilirubinemia in newborns, as well as sepsis (33, 39). A pediatric sepsis educational campaign in Tanzania could be expanded to street billboards, public service announcements, or other methods of information dispersion, but would have to be carefully structured to ensure emergency departments do not become overburdened with children with non-emergent illnesses.

Implementation of an emergency transportation system may also improve mortality outcomes in Tanzania, however, this would be a challenge due to resource limitations. There are currently no ambulatory services provided by the government in Tanzania (40–42). Transportation is sometimes offered for inter-facility transfer and referral, but the ambulances are not staffed by certified health personnel with standardized training (40, 43). Other available ambulances are run by private companies (43), which are inaccessible to most. The implementation of boda-boda ambulances in Tanzania has been suggested as a more affordable solution (44), and there has been success in retention of emergency first aid skills from training programs offered to police and taxi drivers in sub-Saharan Africa (40). A temporary regional pilot program may be able to demonstrate the cost-effectiveness of these life-saving services.

Children who lived in remote regions of Tanzania were less likely to be represented in this study than expected based on population distribution, indicating that they received care elsewhere, were never referred, died before referral or during transportation, or experienced another barrier to reaching MNH. Because MNH is the only public hospital in the country with pediatric critical care, this implies that there may be septic children from more distant regions of Tanzania who are attending hospitals without clinicians trained in acute pediatric care. Increasing the availability of pediatric subspeciality care, such as emergency medicine and intensive care, at regional hospitals throughout the country would likely save lives. Future cohort studies on pediatric sepsis in Tanzania should include hospitals or clinics throughout the country to compare outcomes in septic children across institutions, with and without pediatric emergency or critical care.

SES, as represented by the combined ownership variable, was not associated with delayed presentation in this cohort, which ran contrary to an El Salvadorian study in which children from families with < $2,000 in annual income had a 14-fold increased risk of dying from sepsis (16). Maternal illiteracy, another commonly used proxy for SES, was also significantly associated with delayed presentation to care in this study (16). These findings were not consistent with our results, however, which could be due to the usage of ownership variables as a proxy for SES. Further studies are needed to elucidate which, if any, factors are viable proxies for SES and important in the relationship between SES and timely access to care for septic children in resource-limited settings.

### Limitations

The strengths of this study include its large sample size and 12 month duration, which captured seasonal variation in pediatric sepsis admissions and outcomes at MNH. This contribution to the limited regional data may help increase awareness of pediatric sepsis in East Africa, as well as aid local health workers in risk-stratifying cases for prioritization and resource allocation.

However, this study did have its limitations. For example, non-survivors arrived to MNH with a higher illness severity than survivors, as demonstrated by number of SIRS criteria met, LOD scores, and AVPU categorizations at the time of presentation; however, due to a lack of blood pressure monitoring and biochemical laboratory data, it was not possible to definitively state whether children were in septic shock or multiorgan failure upon presentation. Therefore, heart rates and respiratory rates of survivors and non-survivors were analyzed as clinical indicators of shock (Figures 4, 5).

Another limitation of this study is the significantly larger proportion of non-survivors than survivors with unknown fever durations that could not be classified as delayed or on-time. To ensure the patients lost to follow-up were not significantly different from those who had outcome data, baseline characteristics between these groups were compared and no significant differences were found.

The usage of ownership variables as a measure of SES introduced a limitation to this study, as ownership is just one representation of SES out of many and does not consider other validated measurements, such as income, occupation, and education. A South African study found that ownership of a phone, car, and in-home flush toilet were viable SES measures as they predicted child malnutrition (45), but these findings were not replicated by this study. It is possible that SES was simply not associated with delayed presentation for this cohort, or that these specific ownership variables were not suitable proxies for SES in this setting and context. It is also possible that higher SES was associated with referral or the ability to reach MNH from other regions, because of costs associated with travel and taking time off work for parents or guardians. This would have resulted in underrepresentation of lower-SES families from other Tanzanian regions. Further studies are needed to clarify the relationship between SES and delayed presentation to definitive care for sepsis.

For this cohort, 228 patients were lost to follow-up. Chi-square tests and Wilcoxon rank-sum tests were run to assess significant differences between the full cohort (*n* = 2,031) and only those with outcome data (*n* = 1,803), but none were found, indicating that loss to follow-up most likely did not impact the results of this study (Supplemental Table 1).

## Conclusion

Delayed presentation to definitive care was an independent risk factor for mortality in this cohort, emphasizing the importance of timely presentation to care for pediatric sepsis. In Tanzania, this may be a challenge for families that live in regions of the country distant from Dar es Salaam. Potential interventions include more efficient referral networks and emergency transportation systems to MNH, as well as carefully structured educational programs for pediatric sepsis recognition directed at caregivers. Additional clinics or hospitals with pediatric emergency and critical care may also reduce pediatric sepsis mortality in Tanzania.

## Data Availability Statement

The raw data supporting the conclusions of this article will be made available by the authors, without undue reservation.

## Ethics Statement

The studies involving human participants were reviewed and approved by Institutional Review Boards and Committees on Human Research at Muhimbili University of Health and Allied Sciences (Ref. No. 2016-03-30/AEC/Vol.X/201) and the University of California, San Francisco (IRB # 16-18977, Ref. No. 161295). Written informed consent to participate in this study was provided by the participants' legal guardian/next of kin.

## Author Contributions

TK, HS, BM, and MM: conception and design of the work. TK, HS, and BM: data acquisition. AS: data analysis and first draft of the manuscript. AS, TK, and BM: data interpretation. AS, TK, MM, and BM: manuscript revision and editing. AS, TK, HS, MM, BM, and TR: final approval of the version to be published work and agreement to be accountable for all aspects of the work. All authors contributed to the article and approved the submitted version.

## Funding

Research effort to create this publication was supported by the National Institute of Allergy and Infectious Diseases (award number K23AI144029, TK) of the National Institutes of Health (NIH), the University of California, San Francisco (UCSF) Division of Critical Care, the UCSF Department of Pediatrics Clinical-Translational Pilot grant, and the UCSF Resource Allocation Program Pilot for Junior Investigators in Basic and Clinical/Translational Sciences. The funders had no role in study design, data collection and analysis, decision to publish, or preparation of the manuscript. The views expressed are those of the authors and not necessarily those of the NIH or UCSF.

## Conflict of Interest

The authors declare that the research was conducted in the absence of any commercial or financial relationships that could be construed as a potential conflict of interest.

## Publisher's Note

All claims expressed in this article are solely those of the authors and do not necessarily represent those of their affiliated organizations, or those of the publisher, the editors and the reviewers. Any product that may be evaluated in this article, or claim that may be made by its manufacturer, is not guaranteed or endorsed by the publisher.
