# Supplementary material for: Delayed Presentation and Mortality in Children With Sepsis in a Public Tertiary Care Hospital in Tanzania
Source: Front Pediatr. 2021 Nov 30;9:764163. doi: 10.3389/fped.2021.764163 (PMC8669816; doi:10.3389/fped.2021.764163)
Supplement: Supplementary file 1 [file Data_Sheet_1.PDF]

Supplementary Material

1 Supplementary Data

Supplemental Table 1. Patient characteristics for the full cohort compared to those with outcome data.

Characteristic	Full cohort N=2031	Followed-up N=1803	P-value
**Demographics**			
Age, months, median (IQR)	25.2 (13.1 – 53.9)	24.9 (13.1 - 53.1)	p=0.78
Male sex, n(%)	1158 (57.0)	1033 (57.3)	p=0.89
Regional address, n(%)			
Dar es Salaam	1556 (76.6)	1394 (77.3)	p=0.63
Neighboring regions	272 (13.4)	240 (13.3)	p=0.98
Mid-distance regions	116 (5.7)	97 (5.4)	p=0.71
Far regions	87 (4.3)	72 (4.0)	p=0.71
Malaria positive, n(%)	109/1578 (6.9)	99/1429 (6.9)	p=1.00
HIV positive, n(%)	26/245 (10.6)	22/229 (9.6)	p=0.83
Fully immunized, n(%)	1991/2017 (98.7)	1770/1792 (98.8)	p=0.98
Malnourished, n (%):			
Underweight	524/1813 (28.9)	469/1615 (29.0)	p=0.96
Wasting	382/1487 (25.7)	344/1318 (26.1)	p=0.84
Stunting	634/1734 (36.6)	564/1544 (36.5)	p=1.00
Comorbidities, n (%)			
Anemia	21 (1.0)	20 (1.1)	p=0.95
Asthma	36 (1.8)	31 (1.7)	p=1.00
Cancer	33 (1.6)	28 (1.6)	p=0.96
Cerebral palsy	90 (4.4)	85 (4.7)	p=0.73
Congenital anomalies	16 (0.8)	11 (0.6)	p=0.64
Congenital heart disease	183 (9.0)	173 (9.6)	p=0.57
Diabetes	8 (0.4)	6 (0.3)	p=0.96
Downs syndrome	17 (0.8)	13 (0.7)	p=0.82
Hydrocephalus	25 (1.2)	21 (1.2)	p=0.97
Prematurity	12 (0.6)	9 (0.5)	p=0.87
Renal disease	10 (0.5)	9 (0.5)	p=1.00
Seizure disorder	44 (2.2)	41 (2.3)	p=0.91
Sickle cell anemia	118 (5.8)	110 (6.1)	p=0.76
Tuberculosis	29 (1.4)	25 (1.4)	p=1.00
Other	56 (2.8)	49 (2.7)	p=1.00
**Pre-arrival**			

Fever duration, n (%)			
≤ 48 hours	903 (44.5)	800 (44.4)	p=0.98
> 48 hours	776 (38.2)	704 (39.0)	p=0.62
Unknown	352 (17.3)	299 (16.6)	p=0.54
Antibiotics pre-arrival, n (%)	403/945 (42.6)	352/833 (42.3)	p=0.91
Referred by hospital or clinic, n (%):	947/2028 (46.7)	835/1801 (46.4)	p=0.86
Transportation method, n(%)			
Ambulance	572 (28.2)	517 (28.7)	p=0.75
Bus	904 (44.5)	783 (43.4)	p=0.52
Private car	410 (20.2)	369 (20.5)	p=0.86
Taxi	52 (2.6)	50 (2.8)	p=0.76
Walked	58 (2.9)	55 (3.1)	p=0.79
Other	26 (1.3)	23 (1.3)	p=1.00
Unknown	9 (0.4)	6 (0.3)	p=0.77
**Illness severity**			
No. of SIRS criteria met, n(%)			
Abnormal respiratory rate	1825 (89.9)	1614 (89.5)	p=0.77
Abnormal heart rate	1119 (55.1)	1001 (55.5)	p=0.82
Ill appearing, in distress, not responsive	1228 (60.5)	1115 (61.8)	p=0.40
LOD score, n(%)			
0	1019 (50.2)	893 (49.5)	p=0.62
1	794 (39.1)	717 (39.8)	p=0.69
2	193 (9.5)	171 (9.5)	p=0.71
3	25 (1.2)	22 (1.2)	p=1.00
AVPU scale, n(%)			
Alert	1828 (90.0)	1616 (89.6)	p=0.74
Responds to verbal	32 (1.6)	30 (1.7)	p=0.93
Responds to pain	120 (5.9)	112 (6.2)	p=0.74
Unresponsive	51 (2.5)	45 (2.5)	p=1.00
**Socioeconomic status**			
Maternal literacy, n(%)	1911/2026 (94.3)	1694/1798 (94.2)	p=0.94
Maternal highest education level, n(%)			
No formal school	95 (4.7)	87 (4.8)	p=0.89
Primary school	978 (48.2)	870 (48.3)	p=0.98
Secondary school	495 (24.4)	434 (24.1)	p=0.86
University/ advanced degree	437 (21.5)	388 (21.5)	p=1.00
Unknown	22 (1.1)	24 (1.3)	p=0.58
No. of children <18 years in household, median (IQR)	2 (1-3)	2 (1-3)	p=0.89
No. <5 years, median (IQR)	1 (1-2)	1 (1-2)	p=0.72
Electricity in home, n(%)	1572/2023 (77.7)	1399/1796 (77.9)	p=0.92
Toilet in home, n(%)	1241 (61.1)	1099 (61.0)	p=0.95

Improved water source, n(%)	1937 (95.4)	1715 (95.1)	p=0.77
Private tap	561 (27.6)	505 (28.0)	p=0.82
Public tap or standpipe	1214 (59.8)	1065 (59.1)	p=0.68
Tube well or borehole	257 (12.7)	227 (12.6)	p=0.99
Protected spring	7 (0.3)	7 (0.4)	p=1.00

IQR, interquartile range; Neighboring regions: Pwani, Mjini Magharibi, Unguja, Pemba, Tanga and Morogoro; Mid-distance regions: Arusha, Dodoma, Iringa, Kilimanjaro, Lindi, Manyara, Mtwara and Ruvuma; Far regions: Mbeya, Mwanza, Mara, Njombe, Kagera, Katavi, Kigoma, Geita, Rukwa, Singida, Shinyanga, Simiyu and Tabora; HIV, Human Immunodeficiency Virus; No., number; SIRS, systemic Inflammatory Response Syndrome; LODS, Lambaréné Organ Dysfunction Score; AVPU, Alert-Verbal-Painful-Unresponsive; Comorbidities included anemia, asthma, cancer, cerebral palsy, congenital anomalies, congenital heart disease, diabetes, Down syndrome, Human Immunodeficiency Virus (HIV), hydrocephalus, renal disease, seizure disorders, sickle cell anemia, tuberculosis, and other significant comorbidities
